# Unpredictable, unpreventable and impersonal medicine: global disaster response in the 21st century

**DOI:** 10.1186/s13167-014-0024-9

**Published:** 2015-01-22

**Authors:** Russell J Andrews, Leonidas M Quintana

**Affiliations:** Nanotechnology & Smart Systems, NASA Ames Research Center, Moffett Field, CA USA; Department of Neurosurgery, Valparaiso University Medical Center, Valparaiso, Chile

**Keywords:** Disaster response, Emergency response, Global health care, International medicine, Medical evacuation, Mobile hospitals, Predictive preventive personalized medicine, Trauma, Telemedicine

## Abstract

The United Nations has recognized the devastating consequences of “unpredictable, unpreventable and impersonal” disasters—at least US $2 trillion in economic damage and more than 1.3 million lives lost from natural disasters in the last two decades alone. In many disasters (both natural and man-made) hundreds—and in major earthquakes, thousands—of lives are lost in the first days following the event because of the lack of medical/surgical facilities to treat those with potentially survivable injuries. Disasters disrupt and destroy not only medical facilities in the disaster zone but also infrastructure (roads, airports, electricity) and potentially local healthcare personnel as well. To minimize morbidity and mortality from disasters, medical treatment must begin immediately, within minutes ideally, but certainly within 24 h (not the days to weeks currently seen in medical response to disasters). This requires that all resources—medical equipment and support, and healthcare personnel—be portable and readily available; transport to the disaster site will usually require helicopters, as military medical response teams in developed countries have demonstrated. Some of the resources available and in development for immediate medical response for disasters—from portable CT scanners to telesurgical capabilities—are described. For immediate deployment, these resources—medical equipment and personnel—must be ready for deployment on a moment’s notice and not require administrative approvals or bureaucratic authorizations from numerous national and international agencies, as is presently the case. Following the “trauma center/stroke center” model, disaster response incorporating “disaster response centers” would be seamlessly integrated into the ongoing daily healthcare delivery systems worldwide, from medical education and specialty training (resident/registrar) to acute and subacute intensive care to long-term rehabilitation. The benefits of such a global disaster response network extend far beyond the lives saved: universal standards for medical education and healthcare delivery, as well as the global development of medical equipment and infrastructure, would follow. Capitalizing on the humanitarian nature of disaster response—with its suspension of the cultural, socioeconomic and political barriers that often paralyze international cooperation and development—disaster response can be predictable, loss of life can be preventable and benefits can be both personal and societal.

## Review

### Introduction

#### Problems with current disaster response

Disasters—largely unpredictable, unpreventable and impersonal—take a devastating toll around the world. Since the start of the new millennium, earthquakes alone have claimed upwards of 300,000 lives in each of 2 years (2004 and 2010) and upwards of 100,000 lives in each of 2 more years (2005 and 2008). Cyclones/hurricanes/typhoons claimed upwards of 150,000 lives in 2008, the majority due to Cyclone Nargis, which struck Myanmar (Burma) with approximately 140,000 lives lost [1,2].

**Figure 1:**
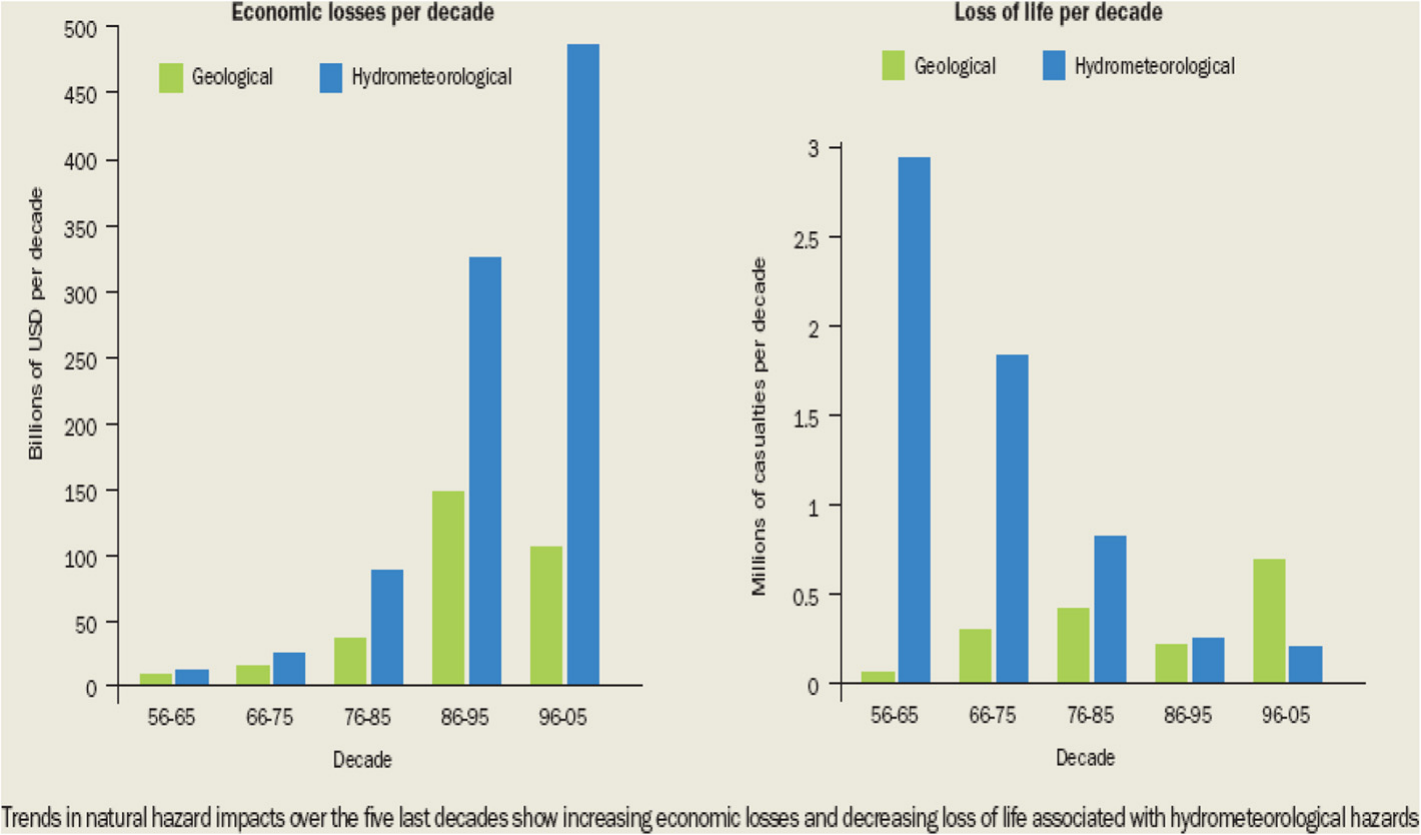
**Economic losses and loss of life from hydrometeorological and geological disasters by decade (from reference** [3] **with permission).**

The impact of natural disasters is substantial, in terms of both economic losses as well as lives lost. The trends for both economic losses and loss of life for the period 1956 through 2005 are graphed by decade in Figure 1. Hydrometeorological causes (notably cyclones/hurricanes/typhoons) have inflicted increasing economic losses (approaching 500 billion USD for the decade 1996–2005), while geological causes (notably earthquakes) have caused increasing numbers of deaths (well over 500,000 for the decade 1996–2005) [3].

The United Nations (UN) has recognized the impact of natural disasters worldwide. In the two decades following the Earth Summit in Rio de Janeiro in 1992, it has been estimated that the damages incurred totaled US $2 trillion and that the number of lives lost was greater than 1.3 million [4]. Following the Indian Ocean earthquake and tsunami that killed upwards of 300,000 people in 2004, the UN World Conference on Disaster Reduction (January, 2005, Kobe, Japan) noted the following [5]:

“We have the knowledge for disaster reduction, what we need is the action. The most important condition for disaster reduction is the political commitment to remove the institutional barriers and integrate disaster risk reduction in the strategies and programmes for sustainable development…”

“We recognize…the importance of involving all stakeholders, including governments, regional and international organizations and financial institutions, civil society, including non-governmental organizations and volunteers, the private sector and the scientific community” [5].

One way to reduce the number of deaths in disasters is to get the medical/surgical “boots on the ground” at a disaster site before the injured have died of potentially survivable injuries. The most dramatic example in recent disasters is the Haiti earthquake of 2010. The Executive Director of Partners for Health, Ophelia Dahl, estimated that upwards of 20,000 people with survivable injuries died every day the first week following the Haiti earthquake because there were no surgical facilities available (to treat fractures, blunt and penetrating trauma, head injuries, etc.) [6]. It is informative that the first and only such international medical response team with surgical capabilities to arrive in Port-au-Prince within 24 h was the Icelandic Association for Search and Rescue, a group always ready for immediate deployment [7].

Why has disaster response been so ineffective in saving those victims with survivable injuries? Some reasons are the following:

1. Disasters typically are relatively rare occurrences in any specific location. Unlike the medical problems addressed by the healthcare system on a day-to-day basis—from chronic diseases such as diabetes, obesity and hypertension to acute events such as pregnancy, motor vehicle injuries and strokes—unless one lives in an earthquake-prone area (e.g. Japan, Chile) or a cyclone/hurricane/typhoon-prone area (e.g. the Caribbean, the Western Pacific), one is unlikely to experience a major disaster on more than a very occasional basis. Healthcare resources are likely to be spent on more frequent (if relatively benign) events such as the common cold, urinary tract infections and pneumonia than on very rare (but usually fatal) events such as Jacob-Creutzfeldt disease, cardiac arrest and the Ebola virus (apart from episodes like the current Ebola crisis).
2. Disasters are usually unpredictable. It is difficult to commit resources to an adverse event that occurs rarely and (given our lack of understanding of the aetiology) seemingly randomly. In the traditional healthcare system, there is, for example, more “bang for the buck” in maternal prenatal care than in screening the population for potential sudden cardiac arrest.
3. Disasters by their very nature evoke a humanitarian response. This falls outside the typical definition of a government’s responsibility to its citizens (security, education, basic health care etc.). Because disasters are unpredictable and unpreventable, they fall “between the cracks” of traditional government agencies. The responsibility for disaster response is often delegated—perhaps “relegated” is more accurate—to religious and/or non-governmental groups (e.g. the Red Cross and Red Crescent, Médecins Sans Frontières).
4. Finally—but likely most importantly—disaster response as currently configured requires the coordination of various government agencies in order to be implemented. The time required for administrative approvals to initiate a disaster response when multiple agencies are involved is incompatible with saving the lives of those who have suffered survivable injuries but who require prompt (i.e. within hours, not days or weeks) medical care.

**Figure 2:**
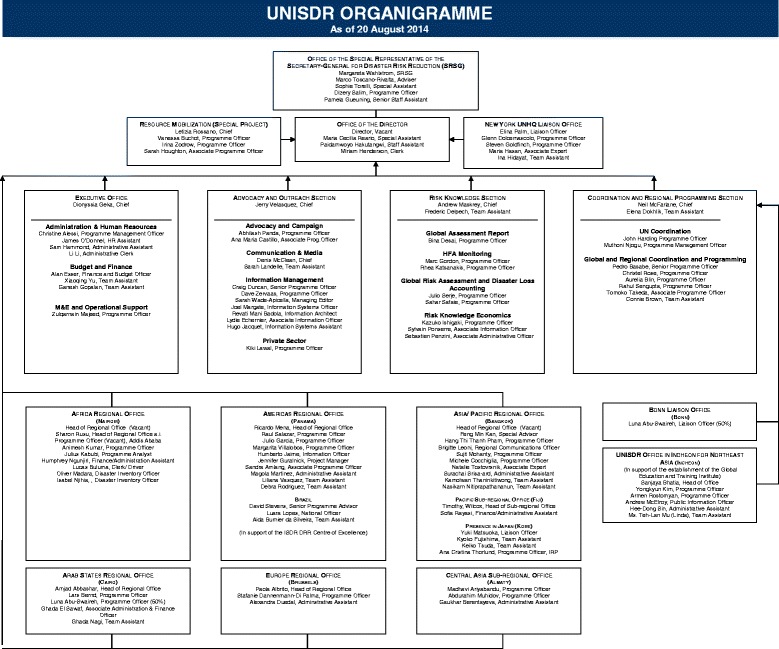
**Organizational chart for the UN International Strategy for Disaster Reduction (from reference** [8] **with permission).** Natural hazards impact trends.

International organizations such as the UN and the World Health Organization (WHO) not only have documented the high cost of disasters—both in economic terms and in lives lost, as noted above—but also have created a multitude of agencies to react to disasters. The United Nations Strategy for Disaster Reduction (UNISDR)—just one of several UN agencies charged with disaster response—has a considerable bureaucracy as evidenced in Figure 2 [8].

The WHO timeline for disaster response, which consists of 23 performance standards, highlights the problem with the current procedures for disaster response. It is not until day 3 (performance standard 7) that “the arrival in-country of a team of experienced professionals” is expected. Moreover, the WHO performance standards are concerned almost exclusively with administrative not medical issues and the timely production of reports rather than the timely saving of lives [9].

An example of administrative bureaucracy paralyzing disaster response comes from Japan (which likely has one of the most robust disaster response programs). In early 2011—before the Fukushima earthquake and tsunami—the Japanese Air Self Defense Force (JASDF) had created what was essentially a three-bed mobile intensive care unit (the Aeromedical Evacuation Squadron—AMES), specifically for events such as the Fukushima disaster. Ironically, between April 2011 and April 2014, the AMES unit was used on only ten occasions. All of these ten missions involved the transport of a single patient (not the three patients the AMES is capable of transporting), none of whom was a disaster victim: the diagnoses ranged from hepatorenal failure to acute cardiac conditions. The reasons cited by members of the JASDF for the failure to use the AMES in disaster response included the following [10]:

1. The prefecture government’s “name recognition” of the AMES availability for disaster response was low.
2. The prefecture government was unable to notify JASDF of the need for the AMES in a timely manner.
3. The need for the JASDF to provide supplies to the disaster site was a higher priority than the use of the AMES for transport of critically injured patients.

Another organization that has provided extensive disaster response in the Asia-Pacific region is the Australian Defense Force Air Medical Evacuation group (ADF AME). Based on over a decade of disaster response missions, the ADF AME suggested the following improvements are needed [11]:

1. A “short notice to move” structure is needed, i.e. rather than responding to each disaster with a “mission”, the AME needs to have an ongoing system in place for immediate deployment.
2. The ADF should be integrated seamlessly with civilian resources for disaster response.
3. Multinational forums and agreements are needed to bring about regional integration of the disaster response teams amongst the various countries in the Asia-Pacific region.

#### Disaster response—the good, the bad and the opportunity

On August 4, 2010—less than 6 months after the devastating earthquake and tsunami that struck south-central Chile—a man-made disaster struck northern Chile: the Copiapó mining accident. Thirty-three miners were trapped 2,300 feet below the surface in the San José copper-gold mine. Seventeen days after the accident, it was discovered that the 33 miners were in fact alive in an underground shelter. The Chilean government’s response included the rapid mobilization of the following resources: virtually every Chilean government ministry, three international drilling rig teams and more than a dozen multinational corporations, as well as the US National Aeronautics and Space Administration (NASA). On October 13, 2010—more than 2 months after the accident—this global rescue effort safely rescued all 33 miners [12].

The comparison of the loss of life from recent cyclones/typhoons in south and southeast Asia is also informative (Table 1). With cyclones/hurricanes/typhoons and similar meteorological disasters, there is—fortunately—more advanced warning than with geological disasters such as earthquakes and volcano eruptions. In Cyclone Nargis, nearly 140,000 lives were lost; in Typhoon Haiyan 7,000; and in Cyclone Phailin, less than 50.

**Table 1:** **Comparison of recent cyclones/typhoons in South and Southeast Asia**

**Name**	**Country**	**Date**	**Maximum wind speed (km/h)**	**Estimated deaths**
Nargis	Myanmar	April 2008	215	~140,000
Phailin	India	October 2013	260	<50
Haiyan	Philippines	November 2013	315	~7,000

Estimated deaths from three recent cyclones/typhoons (from references [13-15] with permission).

What can account for the very high loss of life in Cyclone Nargis and the very low loss of life in Cyclone Phailin? Likely factors in Cyclone Nargis were the failure of the government to provide adequate warning and evacuation of those living in the Irrawaddy Delta, as well as the government’s failure in the early days following Cyclone Nargis to allow international assistance to participate in the disaster response. In Cyclone Phailin, likely the primary reason for relatively little loss of life was the establishment in the Indian state of Odisha of 31 telemedicine stations that very effectively coordinated a heroic evacuation effort: upwards of 1.3 million people were moved to 600 storm shelters. The system for disaster response was already in place and not dependent on the approvals and coordination of various agencies for the response to be implemented.

Although disasters can be devastating, the devastation can be reduced dramatically by effective planning and immediate initiation of the disaster response—as demonstrated by the governments of Chile and India in the examples above. The fact that disasters do not respect the borders between countries, the political differences between governments and the socioeconomic, cultural and religious differences amongst people make disaster response a unique opportunity to improve health care beyond merely reducing the morbidity and mortality of disasters. The need to respond immediately to a disaster eliminates the time for consideration of reasons why one should not do what is right from a humanitarian and moral aspect.

#### Disaster response—Requirements, resources and techniques

Whether natural (e.g. earthquakes, typhoons and tsunamis) or man-made such as terrorist events (e.g. bombings or biosabotage) and large-scale accidents (e.g. airplane crashes or collapsed buildings), disasters not only physically damage large numbers of individuals, but they also damage or destroy the medical infrastructure in the region affected by the disaster. A surgeon without basic imaging/laboratory/blood bank, an operating room and support staff is useless. Not only are medical personnel needed (note that medical personnel in the disaster zone are not themselves immune from incapacitation or death from the disaster), but the medical facilities and infrastructure needed to run the operating room must also be imported.

Some of the equipment needs for disaster response include

- operating rooms (including anesthesia, instruments, sterilization, imaging/laboratory/blood bank)
- electricity (generators)
- food and water
- sanitation and accommodation for both personnel and patients.

Some of the personnel needs for disaster response include

- operating room staff (anesthesiologist, surgeon, nurses, support staff)
- infrastructure staff (imaging/laboratory/blood bank)
- supply teams (transporters, likely helicopter in most situations and logistics)
- social service and rehabilitation (for postoperative care of patients).

In summary, not only are medical resources such as hospitals and doctors, and local infrastructure such as clean water and electricity, likely to be unavailable in a major disaster but the access routes for supplies (notably highways, railways and airports) are likely to be destroyed or unserviceable as well. One must be able to import all necessary resources, usually by helicopter.

**Figure 3:**
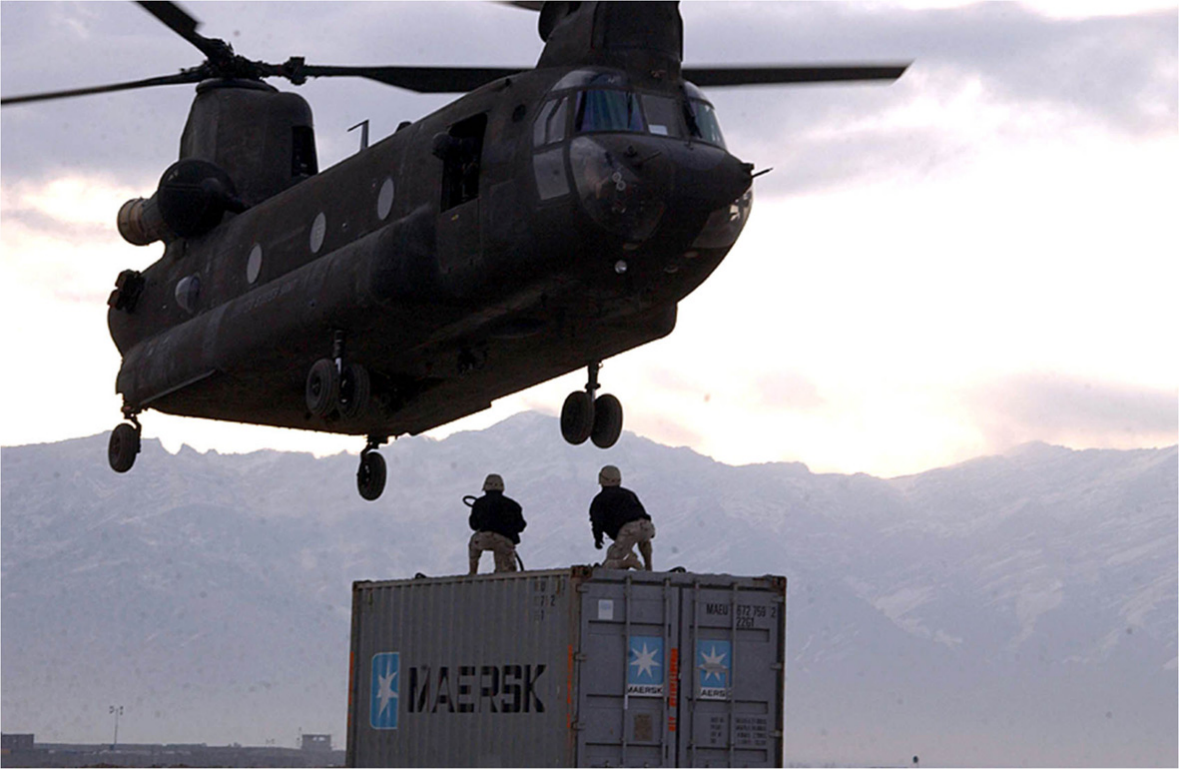
**Chinook transport helicopter—payload 12,000 kg; range 1,100 km.**

Immediate response to medical emergencies has typically been the province of the military in most countries. In the USA, the US Army has developed the mobile emergency unit (MEU), a cargo container that—in various configurations—can serve as a self-contained operating room, recovery room and patient ward. Combined MEUs make up a combat support hospital, which parallels the civilian need for immediate medical resources in a disaster. The MEUs can be transported by helicopter (Figure 3). Other civilian non-governmental organizations such as the International Medical Corps (IMC) have similar self-contained transportable operating room facilities for disaster response.

**Figure 4:**
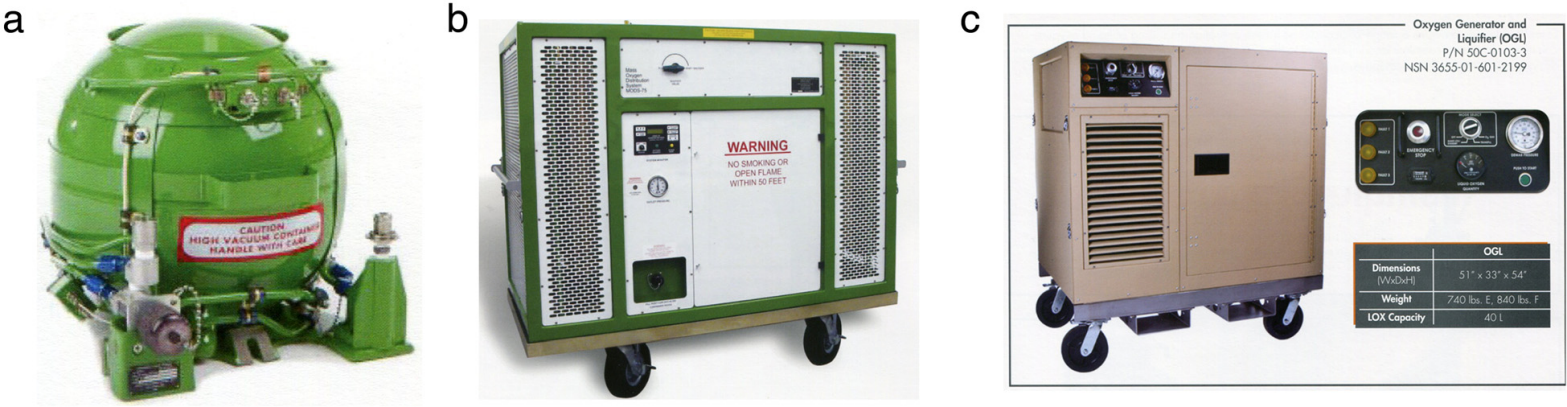
**Lightweight devices for the transport of liquid oxygen and its conversion to gaseous oxygen for patient use. (a)** 10-L Liquid Oxygen Converter (LOX)—full wt 20 kg—up to 8,600 L of gaseous oxygen (courtesy of Essex Industries, St. Louis, MO, USA). **(b)** Mass Oxygen (LOX) Distribution System (MODS)—up to 64,500 L of gaseous oxygen (courtesy of Essex Industries, St. Louis, MO, USA). **(c)** Oxygen Generator and Liquifier (OGL)—generates 1 L of LOX per hour; generated LOX can fill MODS **(b)** (courtesy of Essex Industries, St. Louis, MO, USA).

The recent technological advances in portable medical equipment to support disaster response are impressive. Available presently are lightweight devices for the transport of liquid oxygen and its conversion to gaseous oxygen for patient use (Figure 4a), mass oxygen distribution systems for providing oxygen for up to 150 casualty patients simultaneously (Figure 4b) and liquid oxygen generators for use in harsh remote environments (Figure 4c). For patient monitoring and cardiac defibrillation, small, lightweight devices are available that—in addition to defibrillation—can simultaneously monitor a 12-lead electrocardiogram, noninvasive blood pressure, end-tidal CO_2_, oxygen saturation, two temperature channels and three invasive pressures—with a 6-h battery life.

**Figure 5:**
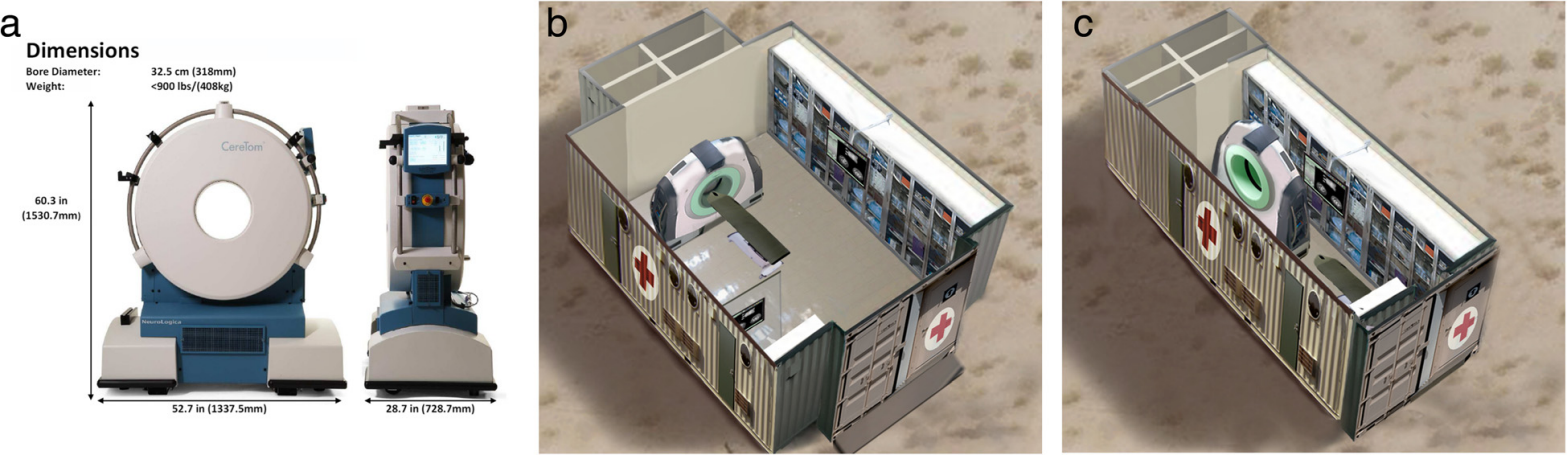
**Computerized tomography scanning in the evaluation and treatment of trauma patients. (a)** Ceretom portable head CT scanner—can run on car battery with inverter (courtesy of NeuroLogica, Danvers, MA, USA). **(b)** BodyTom portable CT scanner in operational mode (portable imaging/operating theatre) (courtesy of NeuroLogica, Danvers, MA, USA). **(c)** BodyTom portable CT scanner in transport mode (portable imaging/operating theatre) (courtesy of NeuroLogica, Danvers, MA, USA).

Computerized tomography (CT) scanning is crucial in the evaluation and treatment of trauma patients. A 400-kg portable head CT scanner (CereTom®) has been developed by NeuroLogica Corporation (Danvers, MA, USA, see Figure 5a). The CereTom CT scanner has all basic capabilities (contrast CT, CT angiography and xenon perfusion CT), can be easily moved on rollers by one person and can be powered by a 12-V car battery using an inverter. A larger but still portable version for body CT scanning (BodyTom®) is also available. Figure 5b, C illustrate the BodyTom CT scanner in both operational (Figure 5b) and transport mode in a containerized imaging room/operating room that can be airlifted to the disaster site (as shown in Figure 3). NeuroLogica Corporation has been acquired by Samsung Corporation, which should result in worldwide availability and support.

**Figure 6:**
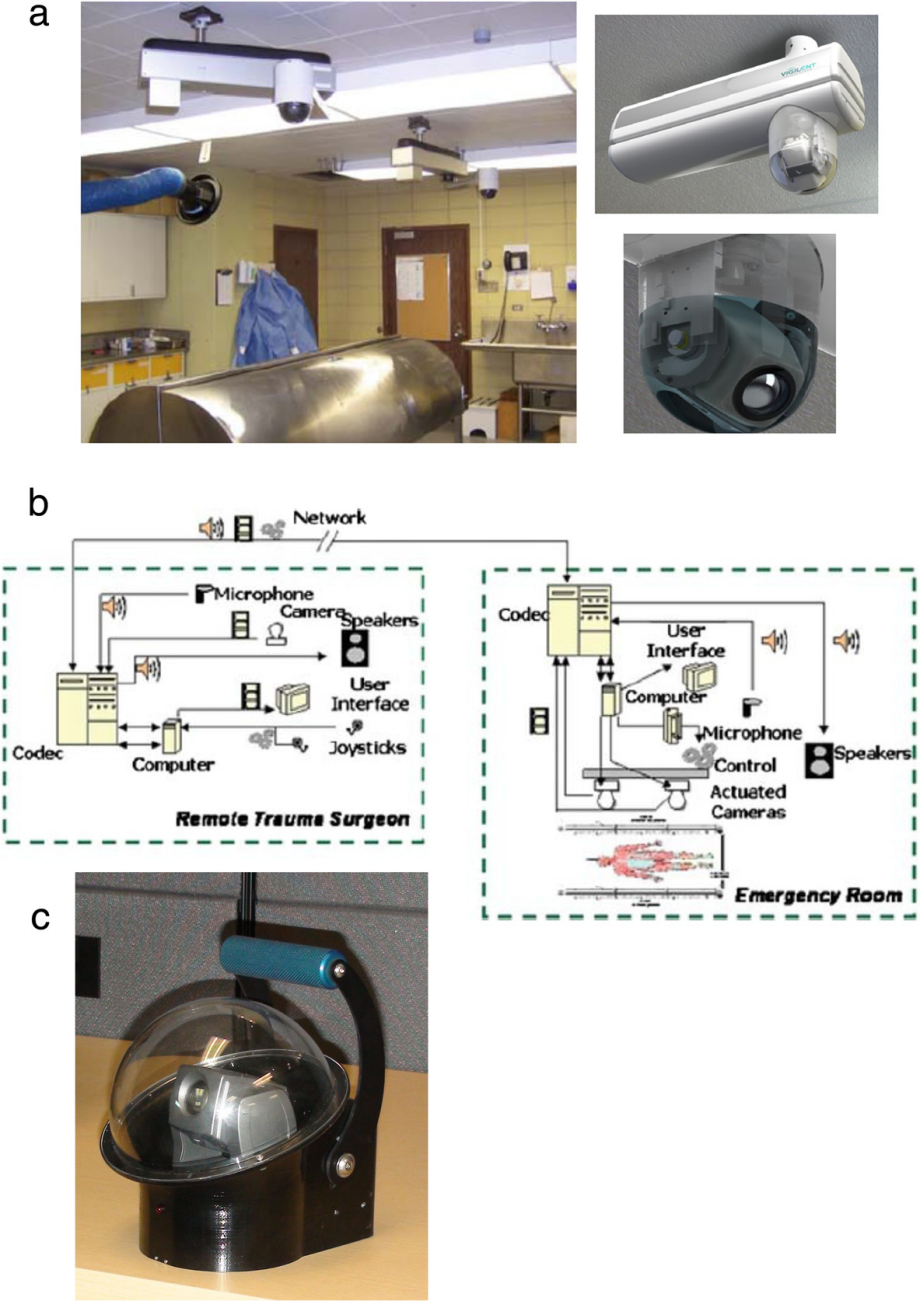
**Remote-control camera system for providing real-time specialist guidance for physicians in remote clinics. (a)** Top—overview of two ceiling-mounted remote-control cameras in emergency room. Bottom left—closeup of mobile arm + camera. Bottom right—closeup of mobile camera. (courtesy of Vigilent Telesystems, Dorval, QC, Canada). **(b)** Schematic of emergency room and remote trauma surgeon for “telesurgery” (courtesy of Vigilent Telesystems, Dorval, QC, Canada). **(c)** Photo of portable, handheld remote-control camera system (courtesy of Vigilent Telesystems, Dorval, QC, Canada).

Frequently, the resource that is most difficult to have immediately at the site of a disaster is the medical/surgical specialist, such as a trauma surgeon, an orthopaedic surgeon or a neurosurgeon. Telesurgery allows the remotely located medical specialist to be “virtually” present at the disaster site. Surgical procedures unfamiliar to the “generalist” physician or the “first responder” emergency team at the disaster site can often be managed if the medical specialist (e.g. trauma surgeon) can act as a “virtual surgical assistant”. Vigilent Telesystems (Dorval, QC, Canada), with the assistance of the Canadian Government, has created a remote-control camera system for providing real-time specialist guidance for physicians in remote clinics in northern Quebec (who may be 1,000 km or more from the nearest major medical center in Montreal or Quebec City). The telesystem consists of two remotely controlled robotic arms, each with a camera, which can be mounted on the ceiling of either a remote clinic or a portable operating room (MEU) (Figure 6a, b). A medical or surgical specialist, perhaps thousands of kilometres away, is able to control the cameras and interact verbally with the medical personnel at the disaster site, much as an attending surgeon might supervise a junior colleague in training. A portable, briefcase-sized, battery-powered, remotely controlled camera is being developed to allow continuous visual (vital sign etc.) monitoring of a trauma patient from the site of injury when the first responders arrive on the scene, in the ambulance or helicopter, to the trauma hospital and the operating room. The remotely located medical/surgical specialist could thus monitor and direct the care of a trauma patient from the scene of injury to definitive in-hospital treatment at either a medical center or a disaster response MEU. Such a portable remote monitoring device is functional wherever there is Internet access.

Perhaps the best example of a major telemedicine program developed with the efficient use of resources in mind is the Apollo Telemedicine Networking Foundation (ATNF), which is part of the Apollo Healthcare System in India. The ATNF president, Krishnan Ganapathy, is a neurosurgeon in Chennai who has spearheaded the development of a telemedicine system not only to support health care throughout India but also to provide teleconsulting services on a daily basis to other countries in the region and additionally to over 20 sub-Saharan African countries [16].

#### Disaster response—lessons from trauma and stroke centers

Decades ago, it was documented that prompt medical/surgical treatment for both trauma and stroke victims resulted in improved outcomes. Thus having hospitals and medical centers with 24/7 availability of the personnel needed to treat trauma and stroke victims (either in-house or on-call for immediate response) became the norm in developed countries. Recent reports confirm the advantage of trauma and stroke centers for patient outcomes. Regarding trauma centers, a recent study considered over 6,000 severely injured motor vehicle accident victims who were initially taken either to a trauma center or to a non-trauma center (and transferred to a trauma center with 24 h for more definitive care) [17]. Nearly half of the victims (45%) were taken directly to a trauma center, and more than half of those taken initially to a non-trauma center were transferred to a trauma center within 24 h (57% of the non-trauma center patients). Those patients who were initially triaged to a non-trauma center had a 30% increase in mortality at 48 h after injury than those who were initially triaged to a trauma center. Regarding stroke centers, a study of all patients in Finland who suffered an ischemic stroke 1999–2006 (more than 60,000 patients) considered whether the patient was treated in a general hospital or in a stroke center (primary or comprehensive) [18]. The case-fatality rate by 1 year for patients treated in a stroke center was less than 18%, while for those treated in a general hospital, it was over 27%; the percentage of patients treated in a stroke center who were home at 1 year was over 73%, while for those treated in a general hospital, it was less than 60%.

What can be learned from the trauma and stroke center systems to improve disaster response? The key to the success of the trauma/stroke center model is that it is fully integrated into the ongoing healthcare system. A trauma or stroke patient is treated in a manner identical to any other patient requiring medical care; there is no separate or parallel delivery system for trauma/stroke care. From medical student education to the rehabilitation phase of patient care, the treatment of trauma/stroke patients—and the trauma/stroke center concept—has been completely integrated into the ongoing healthcare delivery and education system. There is no separate administration or bureaucracy whose approval must be sought before treating a trauma or stroke patient. Clearly, if there were such a separate bureaucracy or authorization process, the resulting delays in care delivery would render useless any potential advantages of having the trauma/stroke medical personnel in place.

#### Disaster response—creating opportunity from unpredictable, unpreventable and impersonal medicine

There are other significant advantages to integrating disaster response into the ongoing healthcare delivery and educations system—beyond the immediate response necessary to achieve improved morbidity and mortality in disaster situations. One of the major goals of many international medical/surgical organizations—organizations often composed of the individual national or regional medical/surgical societies—is the standardization of medical/surgical education and training across nations, as well as the standardization of certification of trainees for licensure in their given specialty. Neurosurgery is a specialty intimately involved in disaster response and will be considered here with regard to benefits beyond improved patient outcomes in disasters. However, the points made below apply to other specialties ranging from emergency medicine to anesthesiology to trauma surgery to orthopaedics, etc.

The primary global neurosurgical organization is the World Federation of Neurosurgical Societies (WFNS), which consists of five continental neurosurgical associations, e.g. the European Association of Neurosurgical Societies (EANS), 114 national neurosurgical societies and five affiliate societies, in total involving 30,000 neurosurgeons worldwide. To quote from the WFNS website [19]:

“The WFNS aspires to promote global improvement in neurosurgical care. The mission of the WFNS is to work together with our member societies to improve worldwide neurosurgical care, training and research to benefit our patients. …The purpose of this Federation shall be the advancement of neurological surgery in all of its aspects by…promoting, implementing and improving minimum and higher standards of neurosurgical care and training worldwide.”

To date, neurosurgical training and certification for practice as a neurosurgeon has taken place at the country level. An exception to this has been the creation by the WFNS of 19 regional international training centers such as the WFNS-sponsored neurosurgical training center for young African neurosurgeons in Rabat, Morocco, that began in 2002.

The EANS has taken the lead in unifying neurosurgical education, training and certification by offering courses that provide continuing medical education throughout the European Union as well as written and oral certification examinations (similar to the US board certification process) that are also recognized throughout the European Union. Individual neurosurgical training programs anywhere in the European Union can apply to become an EANS-accredited neurosurgical training program. A global disaster response system would address one of the primary purposes of worldwide medical/surgical societies such as the WFNS—the establishment of universal high-standard medical/surgical training.

The advantages of such a global disaster response system for the goals of multinational medical/surgical organizations such as the WFNS and EANS include

1. Global standards for medical education and training
2. Global standards for medical certification of competency and licensure
3. The exchange of in-training (residents/registrars) and senior physicians/surgeons that becomes possible with global standards
4. The camaraderie amongst physicians/surgeons worldwide that results from such personnel exchanges
5. The benefits on medical/surgical demography worldwide that result from such personnel exchanges
6. The world-class research opportunities that result from global disaster response—a platform for a global approach to understanding medical/surgical problems ranging from trauma to post-disaster infections and psychological disorders

#### Disaster response—implementing and integrating the DRC into ongoing health care

We have seen that the trauma/stroke center model of integration into the ongoing healthcare delivery and education structure can result in improved patient outcomes in local (and national) trauma/stroke events. “Immediate” is one key word in these programs that require timely response—the equipment and personnel must be ready on a moment’s notice “24/7” to provide care. “Integration” is the other key word in the success of these programs that require timely response—not a response conditional on administrative authorizations by even one agency (let alone the multitude of agencies even in a single country) that are required for a disaster response at present. Eliminating the administrative “middleman” (actually “middlemen”, “middle persons” or “middle bureaucracy” is more accurate) in trauma/stroke centers has saved countless lives over the past several decades. The time has come to apply this model to disaster response—which requires a multinational/regional (and ideally global) integration of the immediate response seen in trauma/stroke centers.

How can such a global disaster response program be initiated? To be effective, a disaster response center (DRC) must be located within a reasonable distance/time from any potential disaster site. Practically speaking for helicopter transport of the portable operating room and other resources, this means within roughly 2,000 km (the range of a transport helicopter with one refuelling stop—in effect about 10 h transport time). This would allow an operating room to be functional at a disaster site within 24 h of a disaster, although with an increasing number of DRCs and improvements—with practice—in the time necessary to set up the operating room and other resources at the disaster site, the time from notification to medical/surgical support should be decreased to 12 h or less.

To be within 2,000 km of most of the world’s population (in regions whose health care can be considered less than “developed”), one would need at least four DRC sites: centrally located in both (1) South America and (2) Africa, as well as (3) Central Asia and (4) Southeast Asia. Two of these regions have advocates who are willing to spearhead an effort to establish their respective DRCs:

1. In Iquique (Northern Chile), the medical center has the support of Leonidas Quintana and his involvement with the Chilean Ministry of Health. Iquique is quite well situated to provide timely disaster response for most of South America.
2. In Peshawar (Northwest Pakistan), Tariq Khan has a particular background and interest in neurotrauma (he has been active in the WFNS Neurotraumatology Committee), has had a well-equipped hospital built in Peshawar and also has involvement with the Pakistani Ministry of Health (including the development of a Trauma Registry for Pakistan). Peshawar is quite well situated to provide timely disaster response for most of Central Asia.

Some of the issues to be addressed for establishing a DRC:

1. Staffing: Ideally, the DRC medical staff (physicians, nurses) is composed of both in-country and out-of-country (likely mostly developed country) staff. Various organizations can provide volunteer physicians from all specialties to staff a DRC, for example, the International Medical Volunteers Association (IMVA), the Health Volunteers Overseas (HVO—US-based), Operation Giving Back (OGB—for surgeons, affiliated with the American College of Surgeons) and the Foundation for International Education in Neurological Surgery (FIENS—for neurosurgeons). For continuity, it may be desirable to emulate the FIENS “Twinning” program—where a university medical center in a developed country partners with a medical center in a developing country for the purpose of providing experienced healthcare personnel as mentors and colleagues over an extended period of time. Another ideal aspect would be for in-training physicians (residents/registrars) from developed countries to receive training at a DRC (for 3 to 12 months), which in a “twinning” situation could involve residents/registrars from the developing country DRC spending an equivalent period at the partner developed country medical center.
2. Licensure and certification: As noted above, within the global neurosurgical community, the EANS has taken the lead in cross-border standardization of licensure and certification in the European Union. A similar worldwide standard for licensure could be implemented for disaster response. Note that such licensure/certification would not be an unrestricted permanent (or renewable) licence to practice medicine in another country—but only a licence to practice during the period of disaster response (and the period when the physician or nurse is in another country for training purposes, e.g. as a volunteer at the DRC).
3. Equipment/devices/drugs/supplies: There are long-term benefits for the companies that support DRCs, from pharmaceutical companies to medical device and equipment manufacturers. Markets for medical equipment and drugs are approaching saturation in developed countries; the ability of a DRC to “fast forward” the development of the healthcare “market” in a developing region will not be overlooked by these companies. Because of the public relations benefits of humanitarian support of a DRC, companies will be eager to donate equipment, devices, drugs and supplies to the DRC, particularly in the initial stages. The favorable tax consequences of such humanitarian medical donations are very persuasive as well.
4. Funding: The out-of-country healthcare personnel (physicians, nurses etc.) would be volunteers, and much of the equipment/devices/drugs/supplies initially provided on a humanitarian basis by medical companies shrewd enough to realize the huge healthcare growth potential in a developing country/region with a DRC. Support would gradually—after, say, the first 2 years—fall increasingly on the host country healthcare system, as the benefits of the DRC for the health of the population in the DRC’s “catchment area” became obvious. Oversight by the involved organizations would establish the milestones for transition to a self-sustaining DRC.
5. Administrative approval or sanction: Once the various organizations agreed on the format and timeline for the implementation of the DRC concept (appropriate branches of the UN and WHO, host country and regional health ministries, international NGOs, international medical volunteer societies and physician/nursing organizations etc.), there would be significant “pressure” on local healthcare authorities to participate productively in the DRC. Although the local healthcare personnel may initially resent the intrusion represented by a DRC integrated into their medical center, the DRC represents a “win-win” for all parties involved. Soliciting local input on the particulars of the DRC in a given locale can be productive from the standpoints of both cost (more economical implementation) and politics (less local resistance to a new program). The benefits of learning efficient healthcare delivery from developing countries (versus assuming the developed countries have knowledge of all the best healthcare policies) have been catalogued by Nigel Crisp (who headed the National Health Service in the UK from 2001 to 2006) [20]. An ongoing, worldwide healthcare project such as the disaster response DRC should not represent a threat to even the most reclusive government. The devastating consequences of Cyclone Nargis in Myanmar in 2008 can be avoided everywhere in the world if disaster response is understood as a global humanitarian effort and not a potential espionage or subversive “photo-op”.

## Conclusions

### Disaster response—benefits of predictable, preventable and personalized disaster medicine

We conclude by summarizing the unique aspects and unique benefits of the disaster response center.

Unique aspects:

1. Like trauma and stroke centers, the DRC is completely integrated into the ongoing healthcare system. The response to a disaster is identical to the response presently in a trauma center when an injured patient is identified or in a stroke center when a stroke patient is identified: the resources and personnel are immediately available to respond to the medical need—without any administrative approvals.
2. The DRC equipment and personnel, being completely integrated into the ongoing healthcare system, serve to augment the local healthcare resources during the non-disaster times. This can make the DRC quite cost-effective, especially given the equipment and personnel that will be forthcoming to create such a valuable healthcare resource for the entire region in the time of disaster and more locally for the other times. This parallels the trauma/stroke center model—where patients in urgent need of the specialized equipment and personnel of the trauma/stroke center are triaged there immediately, while patients with other less intensive medical problems are cared for in centers without such resources.
3. The humanitarian aspect of disaster response will make approaching governments for regional to global cooperation feasible. It will also make donations or discounts of drugs, devices, equipment and supplies from manufacturers more practical. Medical volunteer organizations will have the opportunity to staff simultaneously both ongoing health care in underserved regions as well as disaster response.
4. Telemedicine/telesurgery is an integral part of the DRC concept; the diffusion of telemedicine/telesurgery throughout the world would advance rapidly in support of the DRC mission (during both disaster response and daily healthcare delivery).

Unique benefits:

1. The loss of life including patients with survivable injuries who die from delay of medical/surgical treatment that is routinely experienced today during and immediately following disasters both natural and man-made would be significantly decreased.
2. Daily health care in regions around a DRC would improve dramatically from the infusion of healthcare resources that comes with the DRC.
3. The level of healthcare delivery and medical education would improve in developing countries as they partner with healthcare delivery and medical education in developed countries to staff the DRC. Medical knowledge would flow the other way also, as the healthcare personnel from the developed countries learn techniques regarding efficient and effective healthcare delivery and medical education from their local counterparts [20].
4. Medical education and training, and licensure and certification, would all tend toward a global standard given the cross-border aspects of disaster response and the DRC. International healthcare agencies such as the WHO would assist international medical societies (e.g. for neurosurgery, the WFNS) develop uniform worldwide training and certification standards, using input from member specialists in both developed and developing countries.
5. Regional and global cooperation on disaster response amongst governments that might not be able to agree on many other issues can have, over time, profound effects on breaking down barrier in related areas such as general education (e.g. regional/global standards for high school and university diplomas) and trade (e.g. multinational companies whose products extend beyond health care narrowly defined, such as Johnson & Johnson, Siemens, General Electric, Samsung).
6. The long-term positive effects of camaraderie amongst healthcare professionals from both developed and developing countries working together on a daily basis—especially the junior and in-training personnel who are likely to benefit most from experiencing different social, political and religious points of view—are difficult to overestimate.
7. The DRC will be an unparalleled global research platform to study not only trauma but also related issues ranging from infection and sanitation to rehabilitation and post-traumatic stress.

We conclude with the observation, noted at the outset, by the UN World Conference on Disaster Reduction a decade ago [5]:

“We have the knowledge for disaster reduction, what we need is the action. The most important condition for disaster reduction is the political commitment to remove the institutional barriers and integrate disaster risk reduction in the strategies and programmes for sustainable development…”

Unpredictable, unpreventable and impersonal disasters—anywhere in the world—can be leveraged into a predictable medical response, with preventable consequences on morbidity and mortality and personal benefits far beyond that reduction in individual morbidity and mortality. Integrating disaster response seamlessly into the healthcare training and delivery system worldwide will have socioeconomic effects far beyond the individual lives saved—institutional barriers to universal health care, education and global development will erode in the face of the humanitarian benefits for everyone. The lack of political commitment is not an option—we can no longer afford not to act.
